# Hospital–Provider Company Network for Home Non-Invasive Ventilation: A Feasibility Pilot Study

**DOI:** 10.3390/healthcare12030328

**Published:** 2024-01-26

**Authors:** Michele Vitacca, Giada Asti, Domenico Fiorenza, Gundi Steinhilber, Beatrice Salvi, Mara Paneroni

**Affiliations:** Respiratory Rehabilitation Unit of the Institute of Lumezzane, Istituti Clinici Scientifici Maugeri IRCCS, 25065 Lumezzane, BS, Italy; giada.asti@icsmaugeri.it (G.A.); domenico.fiorenza@icsmaugeri.it (D.F.); gundi.steinhilber@icsmaugeri.it (G.S.); beatrice.salvi@icsmaugeri.it (B.S.); mara.paneroni@icsmaugeri.it (M.P.)

**Keywords:** healthcare, chronic respiratory failure, home, non-invasive ventilation

## Abstract

This study assessed the feasibility of implementing a hybrid hospital–provider company (PC) clinical pathway for patients with chronic respiratory failure (CRF) through the adaptation and follow-up of non-invasive ventilation (NIV). Over a 3-month period, a PC physiotherapist case manager oversaw the adaptation process, making adjustments as necessary, using remote monitoring and home visits. Outcome measures, including the number of patients enrolled, serious adverse events, hospitalizations, survival rates, professional time allocation, NIV adherence, nocturnal apnea–hypopnea, and oxygen saturation, Δ arterial carbon dioxide pressure (PaCO_2_), dyspnea, Short Physical Performance Battery (SPPB), exercise tolerance, quality of life, physical activity, and patient satisfaction, were collected. The recruitment rate was 74% (nineteen patients). Commonly reported adverse events included leakage, discomfort and sleep disturbance. Predominant interventions were four home visits (3; 4) and two NIV adjustments (1; 5). The overall program time commitment averaged 43.97 h per patient (being hospital 40 ± 11% and PC 60 ± 11%). Improvements in PaCO_2_, dyspnea, SPPB and exercise tolerance were observed by the third month. Adherence to NIV was high, with good or very good satisfaction with its use. This study demonstrates that a hybrid hospital–PC service for NIV adaptation and follow-up is not only feasible but also shows validity, reliability, and acceptability.

## 1. Introduction

Long-term home non-invasive ventilation (NIV) represents a therapeutic approach aimed at ameliorating health outcomes by targeting a reduction in carbon dioxide levels in patients diagnosed with Chronic Obstructive Pulmonary Disease (COPD) and those experiencing persistent hypercapnic respiratory failure [1].

While NIV has demonstrated efficacy in reducing hospitalizations, arterial pressure of carbon dioxide (PaCO_2_), dyspnea, and enhancing exercise capacity and health-related quality of life (HRQL) [1], evidence supporting a reduction in mortality remains scant [1]. Uncertainty persists regarding which patient subgroups would derive the greatest benefit from NIV [2].

Historically, logistical considerations for home NIV follow-up and discharge processes have relied on hospital-based monitoring and follow-up, encompassing initiation, adaptation of settings/gear, and either a day-care center or a brief hospital stay without home intervention [3].

Challenges such as limited in-hospital beds, staffing constraints, high hospitalization costs, increased NIV demand, reduced waiting times, patient preferences, and infection transmission concerns [3] have prompted the development of innovative home NIV titration and monitoring settings.

A recent review has underscored the significant benefits and challenges associated with initiating NIV in outpatient or home settings, asserting its safety, feasibility, non-inferiority to in-hospital initiation, and comparable acceptance and adherence rates [3]. The selection of NIV adaptation settings depends on local healthcare structures, legislation, and geographical considerations, acknowledging substantial variations between countries due to differences in healthcare system organization [4].

The emergence of remote ventilator telemonitoring represents a potential paradigm shift in home-assisted ventilation care [5]. Home ventilation devices, such as respiratory assist devices and portable ventilators, now have the capacity to wirelessly transmit usage and performance data to cloud-based web servers for remote access by clinicians, enabling early data review, optimization of ventilatory function through device setting adjustments, and troubleshooting [5].

Existing NIV guidelines [1] offer recommendations on when and how to initiate NIV, but lack specificity on where the ultimate care responsibility should lie—whether public, private, hybrid, hospital-based, or home-based.

This critical knowledge gap, identified as a research priority, necessitates further investigations to determine the optimal setting for NIV initiation, particularly in individuals with chronic respiratory failure (CRF). Additionally, guidelines fail to outline the potential role of private provider company (PC) entities in the adaptation and follow-up of NIV in patients with hypercapnic chronic respiratory failure.

It would be prudent to conduct a feasibility study prior to contemplating the prospective substantial involvement of private entities in the adaptation and monitoring of NIV, whether functioning autonomously or in synergy with public counterparts.

To address this gap, organizational change through a pilot intervention, assessing its feasibility for potential implementation in routine clinical practice, was explored. This study aims to answer the following fundamental questions: Can this new pathway work? Does it work? And will it work? In pursuit of this objective, the initial focus was on appraising the feasibility of a hybrid hospital–PC clinical pathway for NIV adaptation and its subsequent follow-up. This assessment encompassed an evaluation of recruitment capability, resulting sample characteristics, appraisal of data collection procedures, scrutiny of the acceptability of procedures, and an examination of the resources allocated to manage the study.

As secondary objectives, this study aimed to gauge participant responses, focusing on patients’ adherence to NIV protocols, and to assess medium-term clinical and functional changes evident after 3 months of NIV use.

## 2. Materials and Methods

### 2.1. Study Design

This investigation constitutes an observational pilot single-arm study approved by the Ethics Committee (CE2624, 11 May 2022) of Istituti Clinici Scientifici Maugeri IRCCS. This research adheres to the ethical guidelines outlined in the Declaration of Helsinki. Informed written consent was obtained from all participants.

### 2.2. Participants

Conducted at the Respiratory Unit of Istituti Clinici Scientifici Maugeri, Institute of Lumezzane (Lumezzane (Brescia), Italy), this study spanned from June 2022 to August 2023. The facility, a prominent provider of ventilatory support in Lombardy with a population of over 1,266,000 inhabitants, focuses on outpatient care and provides dedicated inpatient beds for complex home ventilation by adhering to prevailing guidelines [1] for respiratory surveillance and decisions regarding the initiation of NIV to alleviate hypoventilation and fatigue by reducing PaCO_2_ levels.

1. Inclusion criteria encompassed consecutive patients diagnosed with chronic respiratory failure (CRF) proposed for the NIV program between 1 June 2022 and 30 August 2023, exhibiting persistent PaCO_2_ values > 46 mmHg within one month post exacerbation/hospitalization. Additional criteria included at least one hospitalization in the prior year and/or a minimum of two severe exacerbations.
2. Exclusion criteria comprised individuals already on NIV, those with significant comorbidities such as congestive heart failure and neurological diseases, pure apnea sleep syndrome, and those unwilling or unable to provide informed consent.

### 2.3. Intervention Programs

A comprehensive home mechanical ventilation (HMV) program was implemented, consisting of the following:

1. Telemonitoring using AirView™ for Ventilation (RESMED, ResMed Inc., San Diego, CA, USA): A cloud-based system facilitating efficient management of patients with respiratory insufficiency. It enables prompt access to patient data, allows the sharing of clinical insights among healthcare professionals, and permits remote adjustments of therapy when necessary. The platform wirelessly connects with ventilators, providing dynamic visualization of therapy data for individual programs.
2. Customizable Care Plans: Tailored plans addressing individual patient needs.
3. Home Visits: Personalized visits to the patient’s residence.
4. Digital Communication: Utilization of messaging and video calls.

Healthcare professionals involved in patient care included hospital staff (physicians, nurses, sleep technicians, and physiotherapists) and the PC service team (physicians, nurses, technicians, and a physiotherapist case manager—PCM).

### 2.4. Hospital Phase

Patients underwent initial adaptation to NIV in hospitals or outpatient settings, facilitated by the Vivisol PC team using a Lumis™ 150 VPAP ST (RESMED, ResMed Inc., CA, USA). The adaptation involved configuring the ventilator, adjusting oxygen levels, and selecting the NIV interface based on patient comfort. If the Fixed Pressure-Controlled (FPC) mode proved ineffective, alternative modalities like average Volume-Assured Pressure Support (AVAPS) were employed.

Comprehensive training on NIV usage was provided to patients during the initial session, with ongoing adjustments to ventilation settings and interfaces guided by the hospital’s physiotherapist under medical supervision. Pre discharge, an educational session covered proper equipment use and care. The patients were registered on the HMV online platform, facilitated by the PC’s technician. All hospital staff were assigned specific duties during the titration phase, as outlined in Table S1 (Supplementary Materials).

### 2.5. Home Phase

The day after discharge, the PCM initiated contact with patients, providing remote monitoring, support, and assistance over three months. During this period, the PCM utilized the following tools:

1. Airview online platform at least three times a week to monitor patients’ progress.
2. Routine calls (every 15 days). Additional calls were conducted in cases of technical requirements or poor adherence.
3. Two mandatory home visits (one in the first week after returning home and one at the end of the 3 months) and extra visits based on patients’ needs (ranging from 1 to 4 visits).
4. Changes to the NIV settings after feedback from the chest physician.
5. Extra instrumental examinations such as arterial blood gases (ABG) and night pulsed oxygen (O_2_) saturation carried out at home by PC nurses and technicians. Final report was filled in by the doctor affiliated with the PC.
6. Regular feedback to the hospital pulmonologist and PC technicians sharing all relevant data.

To address technical challenges or concerns about the provided materials, domiciliary visits were performed by the PC technicians. After the 3-month period, a collaborative pneumological assessment was arranged, incorporating the involvement of the hospital’s attending physician and the PCM. This examination encompassed a comprehensive review of the clinical evaluations conducted, along with an evaluation of the patients’ compliance with NIV.

### 2.6. Measurements

Primary Outcome: Feasibility was assessed through the following:

- Sample characteristics collection;
- Recruitment capability (number of patients included/patients who fulfilled inclusion criteria);
- Acceptability (number of drop-outs, severe side effects within 3 months, hospitalizations, and survival since NIV initiation, and patient satisfaction);
- Evaluation of resources and suitability of procedures (time and dedication by each professional and the mean personnel cost/patient).

Secondary Outcomes: Various measurements were recorded at different time points:

- Only at baseline (T0):

Anthropometric data and clinical history collection.

Spirometry (forced expiratory volume in first second (FEV_1_)% prd, forced volume capacity (FVC)% prd, FEV_1_/FVC, residual volume (RV)% prd, according to the guideline [6].

- At T0, after 1 week (T1) and after 3 months (T3):

Arterial blood gas assessment was conducted with an automated analyzer on blood samples from the radial artery. This assessment was carried out with the individual breathing air or oxygen in a sitting position for at least 1 h. The inspiratory fraction of oxygen (FiO_2_) was calculated from the oxygen flow according to the formula FiO_2_ = 20% + (O_2_ L/min × 4).

- At T0 and T3:

Polysomnography (apnea–hypopnea index/hour (AHI/h), Oxygen Desaturation Index per hour (ODI/h), time spent with oxygen saturation < 90% (T90)) [7].

Dyspnea was assessed with Medical Research Council (MRC) score [8] and Barthel dyspnea (BiD) score [9].

Disability was assessed with Barthel index (BI) [10] and Short Physical Performance Battery (SPPB) [11]; physical activity was evaluated with number steps per day and Physical Activity Scale for the Elderly (PASE) score [12]; and effort tolerance was evaluated with a 6-Minute Walk Test (6MWT) [13].

Quality of life was assessed with the Short Form 12 questionnaire (SF12, with psychological-MCS and physical-PCS items) [14].

- At T1 and T3:

Nocturnal oximetry (ODI/h, T90).

Patient satisfaction in terms of comfort, dryness, leaks, sleep quality, and overall satisfaction (0 = very bad 1 = bad 2 = good, 3 = very good, 4 = excellent).

NIV adherence was calculated as (a) the number of hours per night at each study visit and (b) the percentage of nights with usage exceeding 4 h [15].

- Only at T3:

Patient satisfaction with the entire monitoring service (3 months) (rated from 0 = insufficient to 1 = sufficient, 2 = good, 3 = very good, 4 = excellent).

Number of remote NIV settings changes based on telemonitoring.

Table S1 (Supplementary Materials) summarizes the specific duties of each health professional involved.

## 3. Results

### 3.1. Recruitment Capability

**Figure 1:**
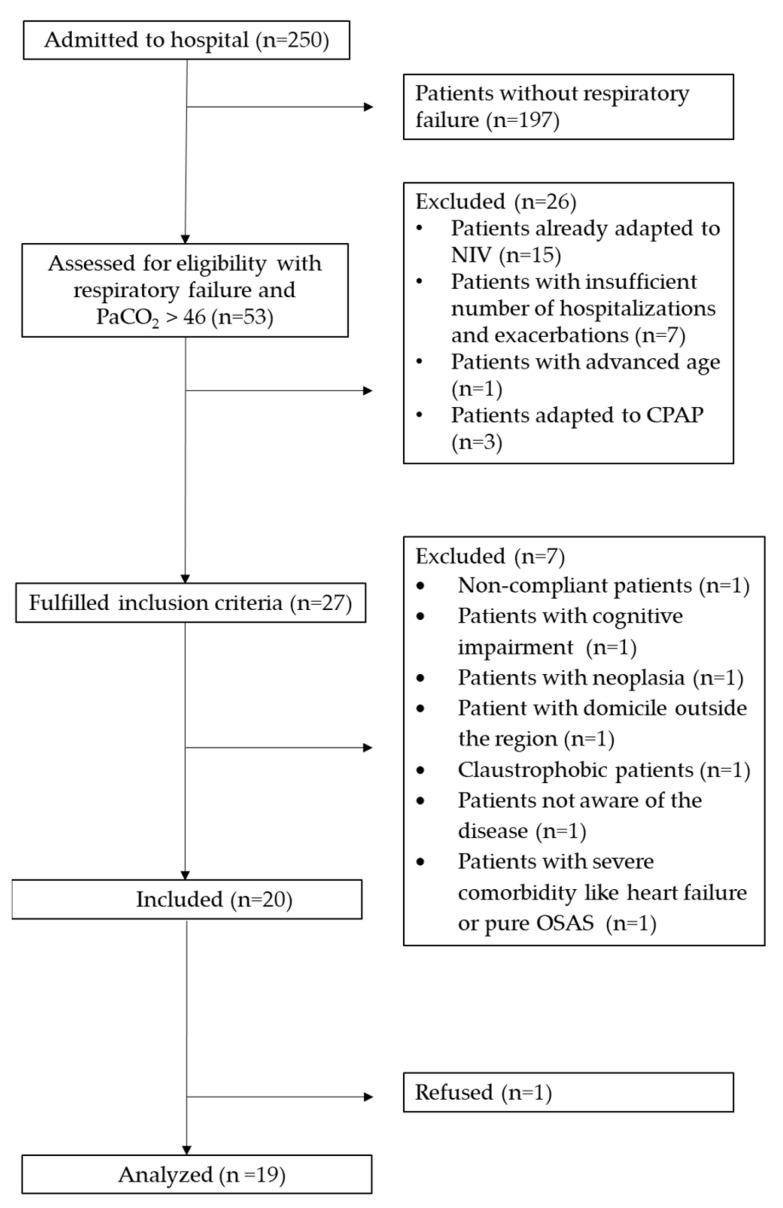
Study trial profile. Legend: paCO_2_ = arterial partial pressure of carbon dioxide; NIV: non-invasive ventilation; CPAP = continuous positive pressure; OSAS = obstructive sleep airway syndrome.

Figure 1 shows the trial profile of the study. Of the total 53 patients who were eligible, 27 satisfied the inclusion criteria, but 7 were excluded for different reasons. Between the first of June 2022 and the last of May 2023, 20 patients (74%) were considered for the program. One patient declined participation after inclusion.

### 3.2. Sample Characteristics

The ultimate analysis relied upon a cohort of 19 patients, and their clinical characteristics are delineated in Table 1. Predominantly, the participants constituted elderly individuals diagnosed with COPD, primarily characterized by an emphysematous phenotype. They manifested severe pulmonary obstruction, diminished exercise tolerance, and stable hypercapnia with CRF, and exhibited symptomatic manifestations including dyspnea. Moreover, the cohort displayed a pronounced clinical history marked by relapses and hospitalizations, diminished levels of physical activity, and discernible declines in both physical and mental dimensions of quality of life.

### 3.3. Procedures

Eighty-four percent of patients (n = 16) underwent NIV titration during hospital admission, whereas the remaining sixteen percent (n = 3) underwent it in an ambulatory setting. The final NIV settings were 18.00 (16.0; 21.0) cmH_2_O for inspiratory positive airway pressure and 7.00 (6.0; 8.5) cmH_2_O for expiratory positive airway pressure.

### 3.4. Acceptability

The most commonly reported side effects associated with NIV are shown in Table S2 (Supplementary Material). Overall, over 40% of patients experienced side effects including leaks, discomfort, disturbed sleep, and morning dyspnea as the main problems. Two patients required hospitalization; however, no deaths were recorded during the 3-month follow-up period.

### 3.5. Suitability of Procedures

Table S3 (Supplementary Materials) displays the number of patients needing extra actions during the protocol. The most commonly performed actions included home visits, NIV adjustment, mask changes, and educational reinforcement. Notably, 95% of patients (n = 18 out of 19) required a median number of four (3; 4) home visits, while 84% of patients (n = 16) needed a median of two (1; 5) remote NIV adjustments.

### 3.6. Resource to Manage

**Figure 2:**
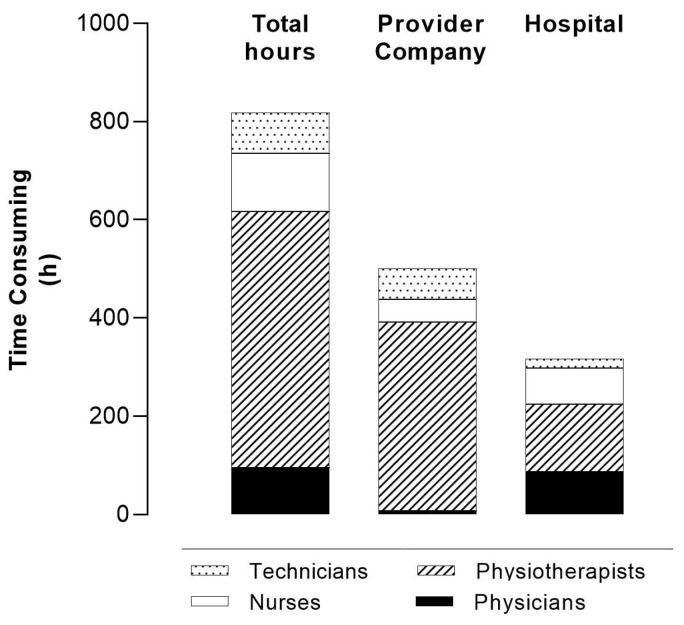
Time consumed (hours) for the global program according to both provider company and hospital.

The overall time taken for the program by all health and technical staff was 43.97 (39.17; 47.55) h per patient (Figure 2), with 17.25 h (15.42; 18.29) and 26.00 h (22.01; 29.74) being the time consumed by the hospital and PC, respectively. Among all the participants, the physiotherapist played the most significant role; doctors and nurses were less involved in the ‘hospital time phase’, while they were involved only marginally in the home phase (Figure 2). The mean personnel cost/patient was 945 ± 142 EUR (ranging from 523 EUR to 1176 EUR), which was 56% and 44% for PC and hospital, respectively.

#### Secondary Outcomes

**Figure 3:**
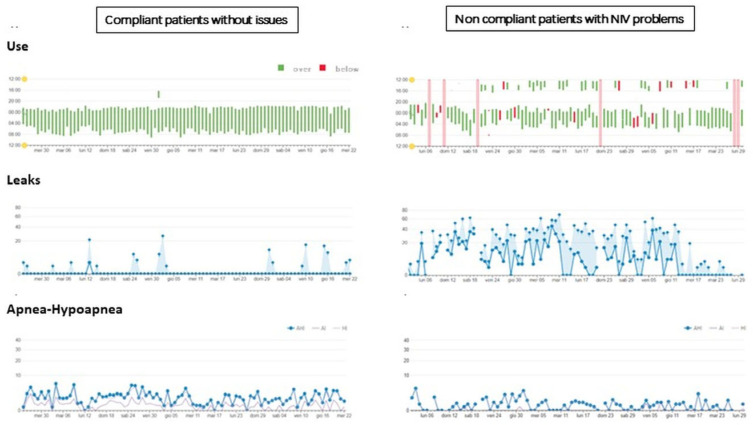
One-month trends for hours of NIV use, leaks, and residual AHI of two representative patients (left panel = a compliant patient; right panel = a non-compliant patient). Legend: NIV: non-invasive ventilation; Panels “Use”: green line corresponds to day with usage ≥ 4 h/day, red line corresponds to day with usage < 4 h/day. Pink line corresponds to missing data. Panels “Leaks”: the bullet points correspond to the median value and square points correspond to 95° percentile. Panels “Apnea-Hypopnea”: AHI: Apnea-Hypopnea Index, AI: Apnea Index, HI: Hypopnea Index.

Figure 3 displays the one-month trends for hours of NIV use, leaks, and AHI in two representative patients. The results demonstrate the utility of web monitoring in distinguishing between screen-compliant patients without issues (as shown on the left side) and non-compliant patients with NIV problems (as shown on the right side).

**Figure 4:**
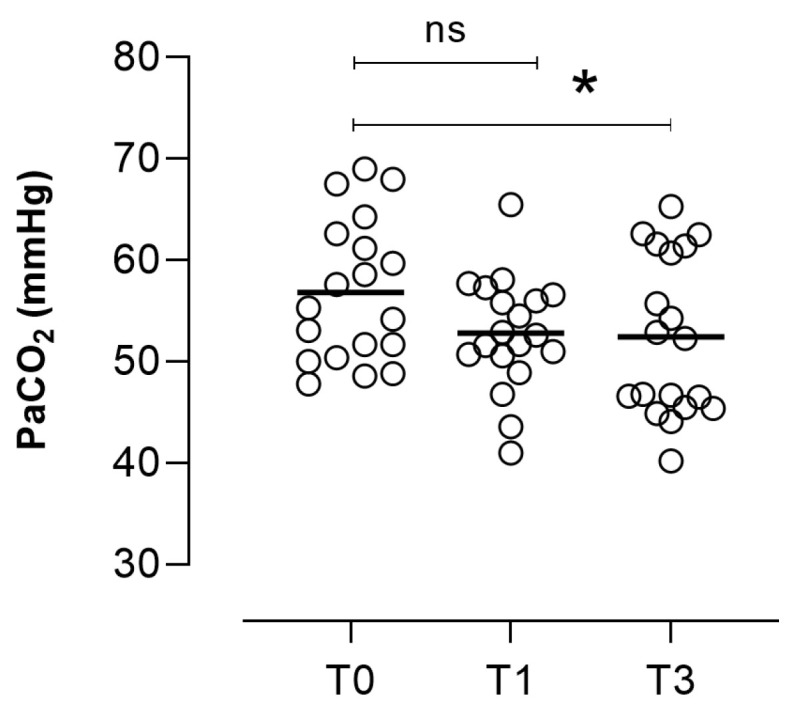
Time course of PaCO_2_ mmHg from baseline (T0) to 3 months (T3) after NIV acclimatization. Legend: NIV: non-invasive ventilation; baseline (T0); T1 = first week and T3 (after 3 months). * T0 vs. T3 *p* = 0.0175; ns = not significant.

The statistically significant time course of PaCO_2_ from baseline to 3 months after NIV acclimatization is presented in Figure 4 (Friedman test, *p* = 0.0132).

The reduction in PaCO_2_ was −4.50 (−8.13; −0.68) mmHg (Table 2: ΔT0 vs. T3 *p* = 0.0235), accompanied by a corresponding improvement of −9.03 (−18.49; −1.03) % compared to baseline. This PaCO_2_ enhancement was evident in 72% of patients, while 22% experienced deterioration. In comparison to the 14 responders, the 5 non-responder patients displayed poorer values for BMI, FEV1% predicted, RV% predicted, and baseline PaCO_2_ and PaO_2_/FiO_2_. Only Barthel dyspnea (*p* = 0.0413) and effort intolerance (*p* = 0.0492) showed statistically significant differences. It is noteworthy that there were no significant differences observed in terms of NIV adherence and the number of home visits.

Beyond the PaCO_2_ improvement, statistically significant enhancements were noted in MRC dyspnea, SPPB, and effort tolerance in the 3rd month (Table 2). Concerning inactivity duration, only seven patients (41%) experienced a reduction, while six patients (35%) displayed an increase in steps/day. Additionally, 71% of patients surpassed the clinically significant minimum improvement of 30.5 m in their 6MWT.

**Figure 5:**
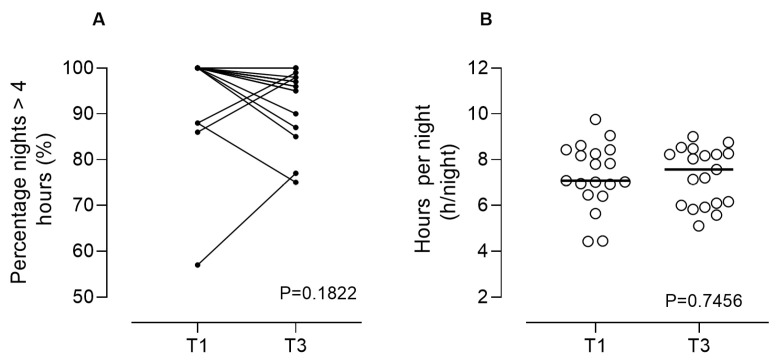
NIV adherence was reported as the percentage of nights with NIV use > 4 h (**A**) and hours per night (**B**) during the home program. Legend: NIV: non-invasive ventilation; T1 (first week) and T3 (after 3 months).

Figure 5 shows that NIV compliance (percentage of nights with more than 4 h of NIV use and total hours per night) was extremely high from the first week of use (T1) for almost all patients. Adherence continued to be high even after 3 months of NIV use (T3).

By the end of the third month, patients reported high levels of comfort with ventilation, scoring a median of 3 (2; 3) for comfort and 3 (1; 3) for relief from dry mouth. They also rated air leakage as good, with a median score of 2 (2; 3), and experienced better sleep quality, scoring 3 (1; 3). Moreover, patients expressed satisfaction with NIV use, rating it 2 (2; 3). Patients gave an overall excellent rating of 4 (4; 4) for the entire monitoring service during the first three months.

After the third month, patients conveyed favorable assessments for various aspects related to ventilation: comfort (median score 3 (2; 3)), dryness (median score 3 (1; 3)), air leakage (median score 2 (2; 3)), sleep quality (median score 3 (1; 3)), and satisfaction with NIV use (median score 2 (2; 3)). Furthermore, the overall monitoring service received an outstanding rating, with a median score of 4 (4; 4), indicating a unanimous excellent rating in patient evaluations.

## 4. Discussion

In this pilot study, the feasibility of a hybrid hospital–provider company clinical pathway for non-invasive ventilation adaptation and follow-up has been demonstrated. The assessment encompassed recruitment capability, sample characteristics, procedures, acceptability, and resource management within the protocol.

### 4.1. Sample Characteristics

Selecting appropriate candidates for NIV initiation is crucial [1,3], especially considering the complexity of severely ill patients. This study underscores the feasibility of proposing and monitoring NIV at home for individuals with advanced age, predominantly COPD of an emphysematous phenotype, and severe lung obstruction. The demographic and clinical characteristics align with established sample profiles in the prior literature and guidelines [1].

### 4.2. Recruitment Capability

The findings from this present pilot study reveal that approximately 21% of admitted patients were potential candidates for NIV at home. However, strict inclusion criteria led to the enrollment of only 50.94% of these individuals. This emphasizes the particular nature of patients requiring NIV at home, constituting a niche with significant resource implications for the payer system. Despite their unique needs, this patient group remains understudied, lacking substantial advocacy for improvements in their care.

### 4.3. Procedures

Following discharge, the HMV center played a crucial role in the complex NIV setup process, requiring multiple adjustments and assessments [16,17]. The present study underscores the importance of a multidisciplinary team approach, involving various healthcare professionals with specific duties (refer to Table S1 in Supplementary Materials).

The diverse practices for initiating NIV reflect the current dearth of research on different care models, influenced by factors such as health system structures, funding mechanisms, historical practices, and local challenges [3].

Traditional hospital initiation of NIV, while historically preferred [1], is perceived as depersonalizing, stressful, and inefficient [18]. The exploration of alternative titration methods outside the hospital setting is driven by factors like patient preference, convenience, and infection transmission risks [18].

Challenges associated with outpatient and home initiation [3] include lack of experience, complications in management, delayed treatment, reimbursement issues, and legal considerations [18].

However, the implementation of an outpatient NIV setup demonstrated comparable effectiveness to inpatient initiation, albeit necessitating multiple hospital visits [19].

Telemonitoring and data transmission have emerged as crucial tools, offering insights into compliance, usage patterns, and potential complications [20,21,22,23].

Notably, more than half of the patients in this pilot study required additional actions during the protocol, including home visits, NIV adjustments, mask changes, and educational reinforcement. Home setup complexities in such patients sometimes necessitate face-to-face home visits for NIV device resets, as observed in the USA [18]. Despite challenges, the collection of essential information such as gas exchange and ventilation data, considered gold standards, was successfully achieved in the present study.

### 4.4. Acceptability

The recent NIV guidelines [1] highlight minor adverse events associated with NIV, and this pilot study confirms that our NIV approach is a safe practice. Over 40% of patients experienced side effects, predominantly leaks, discomfort, disturbed sleep, and morning dyspnea. Two patients required hospitalization, but no deaths occurred during the 3-month follow-up. Common interventions during NIV follow-up included adjustments, mask changes, home visits, and reinforcement of usage. By the third month, patients reported good NIV comfort, minimal air leakage, and satisfactory sleep quality.

### 4.5. Evaluation of Resources

Home mechanical ventilation organizations exhibit significant variability, ranging from private to hospital-based entities [24]. As per a recent survey, the responsibility for long-term ventilator maintenance is assigned to home primary care in 56% of cases, with a notable proportion also managed by prescribing hospitals (26%) or prescribing physicians (16%) [25]. Commercial healthcare providers have endeavored to surmount organizational and financial impediments to implementing home care, particularly in the realm of home ventilation [24]. In select countries, technicians, nurses, and respiratory therapists employed by PCs conduct follow-ups and assessments under the oversight of a hospital team or a pulmonary physician [24]. The organizational structure of healthcare entities [24] may significantly influence whether home follow-up can be outsourced to external healthcare providers. For example, in France, ANTADIR Assistance delivers well-structured home care for respiratory failure treatments, leveraging external care providers and adhering to stringent legal obligations [26]. Similarly, in Israel, home ventilation relies on a specialized home care provider organization, deemed advantageous in terms of both economic benefits and quality of life [27].

The current study proposes a shift in responsibilities from the hospital to a private company, establishing a joint venture. The redistribution of competencies allowed a more balanced contribution to the total time spent on NIV adaptation and monitoring, with physiotherapists playing a central role. During the entire study, the hospital health team maintained close control and coordination of the PC activities and needs. The time consumption and costs associated with the employment of health and technical staff for the entire program were deemed acceptable and reasonable, with huge variability among patients. The cost–benefit ratio warrants individual evaluation based on local realities, organizational structures, and personnel costs.

### 4.6. Outcome Measures

The primary short-term goal of home NIV is to improve carbon dioxide levels [1], and this pilot study shows positive outcomes in blood gases, dyspnea, disability, and effort tolerance. While recent guidelines [1] highlight limited effects on mortality or hospitalizations and a limited effect on arterial partial pressure of carbon dioxide (paCO_2)_, higher effects on PaCO_2_ were found when a very high level of inspiratory positive airway pressure was used, along with a decrease in dyspnea and an improvement in exercise capacity and quality of life. Thus, the present study indicates noteworthy improvements with optimal NIV adherence.

### 4.7. Limitations

Due to the preliminary nature of this pilot study, certain feasibility aspects such as retention, logistical challenges, direct and indirect costs, and implementation of the new care model were not fully evaluated. Additionally, the relatively small sample size and monitoring time are acknowledged limitations.

### 4.8. Clinical Implications

The promising preliminary results indicate that the proposed intervention, aiming to shift a portion of the workload from the hospital to the private sector, is poised for examination in a more extensive trial. This model, favorably received by patients, holds the potential for mitigating hospital burden and inconvenience, especially for individuals residing in challenging geographical areas.

Furthermore, it is imperative to delve into the perspectives of diverse stakeholders who will play influential roles and be impacted by this refined intervention. Addressing potential challenges, such as the hypothetical failure of the private sector, its exclusive prioritization of profit, or the failure to meet adequate care standards, remains crucial. The author posits that any collaborative effort should be orchestrated under the guidance of hospital staff expertise, serving as a safeguard for the health payer.

It is vital to thoroughly explore the viewpoints of various stakeholders and ensure the coordinated implementation of such joint ventures under the supervision of hospital staff, thereby securing the health payer’s assurance.

## 5. Conclusions

Based on this pilot study, the hybrid hospital–provider company pathway for NIV adaptation and the 3-month follow-up demonstrate feasibility, validity, reliability, and acceptability, leading to notable clinical and functional improvements. A larger, controlled study with increased rigor is warranted to further assess standards, reimbursements, and long-term efficiency and cost-effectiveness.

## Figures and Tables

**Table 1:** Patient characteristics at baseline.

Patients, number	19
Age, years	71.0 (67.0; 76.0)
Gender, number (%)	
Male	8 (42.1)
Female	11 (57.9)
Diagnosis, number (%)	
COPD with emphysematous phenotype	8 (42.1)
Bronchiectasis	3 (15.8)
Overlap Syndrome	5 (26.3)
Restricted dysventilation + OSAS	3 (15.8)
BMI, score	20.5 (18.0; 25.0)
Hb, g/dL	12.2 (8.6; 14.5)
CIRS S.I., score	1.8 (1.6; 2.4)
CIRS C.I., score	3.0 (2.0; 6.5)
FEV_1_, % prd	30.0 (19.0; 38.5)
FVC, % prd	52.0 (43.5; 66.5)
FEV_1_/FVC, score	35.6 (31.9; 63.3)
RV, % prd (n = 16)	195.0 (153.0; 247.0)
Patients hospitalized in the previous year, number (%)	12 (63.2)
Patients relapsed in the previous year, number (%)	19 (100)
Number of relapses in the previous year, number	2.0 (1.0; 2.0)
History of ICU admission, number (%)	4 (21.0)
Recent relapse within 3 months, number (%)	6 (31.5)
Drug therapy, number (%)	
LABA + ICS	1 (5.3)
LABA + LAMA	2 (10.5)
TRIPLE	15 (78.9)
SABA + ICS	1 (5.30)
Oxygen, L/min	1.0 (1.0; 2.0)
PaCO_2_, mmHg	55.3 (51.0; 61.9)
pH, score	7.4 (7.4; 7.4)
PaO_2_/FiO_2_	239.0 (210.0; 269.0)
HCO_3_^−^, mEq/L	31.9 (29.2; 34.2)
AHI/h, number (n = 16)	2.2 (0.7; 6.2)
ODI/h, number	4.1 (1.2; 6.7)
Mean SatO_2_, %	94.0 (87.8; 96.5)
T90, %	2.4 (0.0; 83,1)
Barthel dyspnea, score	31.0 (16.5; 40.0)
MRC, score	4.0 (3.0; 4.0)
Barthel index, score	100.0 (91.5; 100.0)
SPPB, score	8.0 (6.5; 10.0)
MCS-12, score	47.7 (42.2; 57.1)
PCS-12, score	30.2 (26.3; 35.6)
PASE, score	136.0 (55.0; 157.9)
6MWT, meters	212.5 (165.0; 288.7)
Steps/day, number	1356.0 (634.7; 2873.5)
Time of inactivity, %	79.5 (69.0; 87.2)
Initial NIV setting IPAP, mmHg	16.0 (13.0; 17.5)
Initial NIV setting EPAP, mmHg	7.0 (5.0; 8.0)
Initial NIV setting Respiratory rate, a/m	12.0 (12.0; 12.0)

Legend: Data are expressed as number, percentage, median, and interquartile range. COPD = Chronic Obstructive Pulmonary Disease; OSAS = obstructive sleep apnea syndrome; BMI = body mass index; CIRS S.I. = Cumulative Illness Rating Scale Severity Index; CIRS C.I. = Cumulative Illness Rating Scale Comorbidity Index; FEV_1_ = forced expiratory volume in the 1st second; FVC = forced vital capacity; RV = residual volume; ICU = Intensive Care Unit; LABA = long-acting beta agonist; ICS = Inhaled Corticosteroids; LAMA = long-acting muscarinic Antagonist; SABA = short-acting beta agonist; PaCO_2_ = Partial Pressure of Carbon Dioxide in Arterial Blood; PaO_2_/FiO_2_ = Partial Pressure of Oxygen/Fraction of Inspired Oxygen; HCO_3_^−^ = bicarbonate; AHI/h = apnea–hypopnea index per hour; ODI/h = Oxygen Desaturation Index per hour; T90 = percentage of time with oxygen saturation below 90%; MRC = Medical Research Council; SPPB = Short Physical Performance Battery; MCS-12 = Mental Health Component Summary of Short-Form Health Survey; PCS-12 = Physical Component Summary of Short-Form Health Survey; PASE = Physical Activity Scale for the Elderly; 6MWT = 6-Minute Walk Test; NIV = non-invasive ventilation; IPAP = inspiratory positive airway pressure; EPAP = expiratory positive airway pressure.

**Table 2:** Changes were observed after 3 months of NIV use for the studied variables.

	∆T3–T0 (CI 95%)	*p*-Value (Wilcoxon Test)
PaCO_2_, mmHg (n = 18)	−4.5 (−8.1; −0.7)	0.0235
PaO_2_/FiO_2_ (n = 18)	21.0 (−34.0; 59.5)	0.2145
HCO_3_^−^, mEq/L (n = 18)	1.1(−3.3; 4.2)	0.6012
AHI/h, number (n = 14)	−0.6 (−3.6; 0.3)	0.1317
Average SatO_2_, % (n = 11)	2.3 (0.7; 8.3)	0.0553
T90, %	−1.2 (−89.1; 0.9)	0.0893
Barthel dyspnea, score	0 (−12.0; 11.0)	0.8245
MRC, score	−1.0 (−1–0; 0.0)	0.0017
Barthel index, score	0.0 (0.0; 1.0)	0.4037
SPPB, score	2.0 (0.0; 2.5)	0.0185
MCS-12, score	3.2 (−5.8; 9.2)	0.3144
PCS-12, score	−1.2 (−3.2; 2.8)	0.8092
PASE, score	−5.0 (−24.4; 27.9)	0.9679
6MWT, meters (n = 17)	70.0 (30.0; 110.0)	0.0021
Steps/day, number (n = 17)	−168.0 (−415.0; 44.0)	0.1024
Time of inactivity, % (n = 17)	0.0 (−4.0; 3.0)	0.9621

Legend: PaCO_2_ = Partial Pressure of Carbon Dioxide in Arterial Blood; PaO_2_/FiO_2_ = Partial Pressure of Oxygen/Fraction of Inspired Oxygen; HCO_3_^−^ = bicarbonate; AHI/h = apnea–hypopnea index per hour; SatO_2_: oxygen saturation; T90 = percentage of cumulative sleep time with oxygen saturation below 90% in total sleep time; MRC = Medical Research Council; SPPB = Short Physical Performance Battery; MCS-12 = Mental Health Component Summary of Short-Form Health Survey; PCS-12 = Physical Component Summary of Short-Form Health Survey; PASE = Physical Activity Scale for the Elderly; 6MWT = 6-Minute Walk Test.

## Data Availability

Data are available upon request to the corresponding author.

## Supplementary Materials

The following supporting information can be downloaded at https://www.mdpi.com/article/10.3390/healthcare12030328/s1. Table S1. Specific duties for each health professional involved. Table S2. Most frequently reported side effects related to the use of noninvasive ventilation during the study. Table S3. Patients needing extra actions during the study.
