# Supplementary material for: Hospital–Provider Company Network for Home Non-Invasive Ventilation: A Feasibility Pilot Study
Source: Healthcare (Basel). 2024 Jan 26;12(3):328. doi: 10.3390/healthcare12030328 (PMC10855547; doi:10.3390/healthcare12030328)
Supplement: Supplementary file 1 [file healthcare-12-00328-s001.zip › healthcare-2820657-supplementary.pdf]

Hospital - Provider Company network for home noninvasive ventilation:

A feasibility pilot study

Supplementary materials

Table S1. Specific duties for each health professional involved.

Figure	Actions
**Physician**	
Hospital	Identify patients suitable for the protocol
	Provide explanations through interviews regarding Non-Invasive Ventilation (NIV) treatment to patients and their family members
	Supervision of adaptation progress
	Check blood gas analysis execution both at T0 and T1
	Prescription of ventilator and mask
	Execution of pulmonary check-up at T3
Provider Company	Reports execution of clinical tests performed at home at T3
**Nurse**	
Hospital	Assistance during the first night of adaptation
	Blood gas analysis and spirometry execution
	Check baseline exam execution
Provider Company	Blood gas analysis home executions
**Technician**	
Hospital	Execution and download of oximeters and polysomnography at T0 and T1
Provider Company	Insertion of the patient to the online platform
	Execution of home visits for technical problems
	Execution and download of home polysomnography at T3
**Physiotherapist**	
Hospital	Initial patient assessment, signature of informed consent and administration of questionnaires both at T0 and T3
	Execution of 6MWT and delivery of the pedometers
	Collecting anamnestic data and information related to the patient's clinical history
	Execution of patient adaptation (under medical indication)
	Clinical monitoring of the patient through data download and execution of oximetry with module
	Implement patient education sessions to teach correct ventilator usage and proper disinfection methods
	Communication with the Service Company (calls and e-mails)
Provider Company	Monitor of the patient over three months through an online platform
	Perform periodic calls and at least 2 home visits plus extra calls and visits to provide patient's assistance and resolution of problems
	Administer questionnaires, delivery and collection of the pedometer, and execution of 6MWT at T3
	Organization of home examinations by intervention of technicians and nurses from the service company
	Provide the doctor a constant update over 3 months about the patient's clinical conditions
	Take part in T3 visit

Legend: NIV= Noninvasive Ventilation; T0= Baseline or recruitment phase before NIV adaptation; T3= 3 months after adaptation; T1= 1 week after adaptation; 6MWT=6-Minute Walk Test.

Table S2. Most frequently reported side effects related to the use of noninvasive ventilation during the study.

Unintentional leaks (around the interface or through the mouth), n (%)	**11 (58)**
Leak-induced xerostomia or dryness, n (%)	**10 (53)**
Leak-induced xerophthalmia or conjunctival irritation, n (%)	**9 (47)**
Pain caused by headgear or mask, n (%)	6 (32)
Skin lesions, pressure sores, and ulcerations, n (%)	3 (16)
Nasal obstruction, congestion, rhinorrhea, nasal mucosal, n (%)	4 (21)
Discomfort, n (%)	**9 (47)**
Abdominal distention and pain, n (%)	3 (16)
Inappropriate alarm settings, n (%)	0 (0)
Perceived patient-ventilator asynchrony (delayed triggering, premature or delayed cycling, auto triggering, n (%)	6 (32)
Disrupted sleep n (%)	**8 (42)**
Claustrophobia or anxiety related to interface, n (%)	7 (37)
Epistaxis, n (%)	1 (5)
Morning dyspnea, n (%)	**9 (47)**

Legend: Data are expressed as number of patients (%); in bold, side effects reported by >40% of patients

Table S3. Patients needing extra actions during the study.

**At distance**	
NIV adjustment during time, n (%)	16 (84)
Different masks rotation test, n (%)	11 (58)
Reinforcement for usage time < 4 hours, n (%)	9 (47)
Mask change, n (%)	6 (32)
Reinforcement because NIV was not used, n (%)	5 (26)
Reevaluation because AHI> 15/h, n (%)	4 (21)
**At home**	
Home visits request, n (%)	18 (95)
Technical home visit due to water in the circuit or tubing assembly problems, n (%)	6 (32)
Technical/Physiotherapy home visits due to technical problems with ventilator, n (%)	4 (21)
Home $pSatO_2$ tracing monitoring request	4 (21)
Change mask break, n (%)	4 (21)

Legend: NIV= Noninvasive Ventilation; $pSatO_2$= Pulse Oximetry Saturation; AHI/h= Apnoea Hypopnea Index per hour.
